# The role of the minimally invasive surgery in the management of paediatric liver tumours

**DOI:** 10.3332/ecancer.2025

**Published:** 2025-11-13

**Authors:** Alyssa Stetson, Gloria Gonzalez, Greg M Tiao

**Affiliations:** 1 Department of Paediatric General and Thoracic Surgery, Cincinnati Children’s Hospital Medical Center, USA; 2 Department of Paediatric General and Thoracic Surgery, Lerner College of Medicine and Case Western Reserve University Cleveland Clinic Children's Hospital, USA; 3 Department of Surgery, University of Chile

**Keywords:** paediatric, liver cancer, minimally invasive surgery

## Abstract

Minimally invasive surgical techniques are increasingly adopted for the management of hepatic masses in children. Laparoscopic liver biopsy can be used to obtain tissue diagnosis while avoiding the risks of open surgery and providing improved cosmesis. Laparoscopic or robotic liver resection has more gradually been adopted in children than in adults but can be utilized for appropriately located tumours as long as oncologic principles are maintained. Patient size is a factor when choosing whether to perform liver resection via a minimally invasive approach. Laparoscopic radiofrequency ablation offers an alternative strategy to surgery for paediatric patients with small masses or can serve as a bridge to transplant.

## Introduction

Minimally invasive surgery (MIS) has become a standard option in the treatment armamentarium for children who have conditions that require surgical intervention. Its role in paediatric oncologic conditions is evolving. In this review, we summarize how MIS is utilized in the management of paediatric hepatic tumours.

## Minimally invasive procedures for liver biopsy

### Introduction

Liver biopsy remains the gold standard for the diagnosis of hepatic pathologies, including masses [1]. Minimally invasive procedures allow for tissue diagnosis without the risks of an open approach.

### Indications

Because of the concern for malignancy, many children who present with a liver mass require biopsy [2, 3]. A biopsy is recommended for children with a liver mass that is not amenable to primary resection.

### Contraindications

Most cases of hepatocellular carcinoma (HCC) in children arise *de novo*, in which case biopsy is still warranted [2]. Nodules arising in a background of cirrhosis that are radiographically consistent with HCC should be treated as such, as biopsy carries up to a 5% risk of needle track seeding. Other possible contraindications to percutaneous biopsy include bleeding disorder, morbid obesity, vascular lesion, extrahepatic biliary obstruction and presence of cholangitis [2].

### Surgical approach

Minimally invasive modalities include percutaneous and laparoscopic approaches [3]. If feasible, percutaneous liver biopsy is the preferred technique due to its safety and simplicity, as well as reduced adhesions between the tumour and abdominal wall [4]. It can be performed using ultrasound or computed tomography guidance [5, 6]. While more invasive, laparoscopic-assisted core needle biopsies allow for visualization of the peritoneal cavity and lesion, increased sample volume and easier control of bleeding [2, 7]. Needles used for biopsy include ‘suction’ needles and ‘cutting’ needles, with the need to balance the safety and smaller size of the suction needle with the superior preservation of tissue architecture using the cutting needle [7].

### Tips and pitfalls

Recent literature indicates that open biopsy and laparoscopic biopsy have higher rates of post-procedural bleeding requiring a transfusion than percutaneous biopsy, but all three approaches carried similar rates of relapse [4]. Risk of bleeding after percutaneous biopsy ranges from 0% to 7.1% and appears to be higher in patients undergoing biopsy for oncologic purposes [8–11].

Core needle biopsy should be performed using a 15-gauge sheath through which a 16-gauge needle can be passed at least seven times and repositioned to biopsy various areas of the mass as well as normal liver parenchyma [12]. Tract embolization should be performed at the conclusion of the procedure. The biopsy tract should be included in the eventual resection specimen to minimize the risk of recurrence due to seeding of the tract [2, 12].

## MIS for liver resection

### Introduction

Laparoscopic liver resection (LLR) was first established in the adult population. It was initially utilized for benign diseases in which the liver lesion was easily amenable to resection, i.e., a wedge resection or left lateral sectionectomy [13]. The results from these early efforts reported decreased morbidity and pain, shorter length of stay (LOS), less estimated blood loss and improved cosmesis [14, 15]. As such, LLR became a viable option for benign lesions [15]. As experience with hepatic MIS grew and laparoscopic devices for transecting parenchyma improved, LLR was trialed for malignant disease [16, 17]. Subsequent studies have confirmed that, in appropriately selected patients, there are equivalent or superior oncologic outcomes between LLR and open hepatic resection [15, 18–20]. It is now recognized as an acceptable approach in select adult patients to treat HCC and colorectal liver metastases with benefits that include the avoidance of a large incision and lower rates of postoperative liver failure and ascites [21].

In the paediatric patient population, MIS hepatic surgery has been more gradually adopted. This can be attributed to the infrequent incidence of paediatric hepatic neoplasms, size limitations of paediatric patients with the attendant need for smaller instruments, learning curve for MIS procedures and start-up costs associated with introducing a MIS program [17, 22–24].

Despite these challenges, LLR is now performed at select paediatric centers for both benign and malignant lesions with studies reporting a similar postoperative complication rate to open liver resection, infrequent need to convert to open for bleeding control and overall oncologic success (Table 1) [14, 22, 23–27]. Widespread adoption remains challenging [28].

### Indications

Indications for LLR in children parallel those for open hepatectomy. Type of resection and adequacy of post-operative functional liver parenchyma overarch decision-making [25, 29].

### Contraindications

*Patient size -* Patient size is a concern due in large part to the dimensions of the current instrumentation [17, 23, 30]. Literature is limited and conflicted regarding how patient size should be incorporated into decision-making, with small series defining weight range and age [23, 25]. A critical determinant are the instruments that make parenchymal transection safer, like the laparoscopic cavitron ultrasonic surgical aspirator (CUSA) device, ultrasound and 12 mm staplers, all of which require larger ports placed with adequate distance to allow the head of the device to be used within the smaller abdominal domain of a child [23].

*Technical safety -* An important consideration for LLR is the management of bleeding [25]. In adults, approximately 6% of LLR are converted to open for bleeding with varying conversion rates based on the type and extent of resection [31–34]. The best way to manage this risk is with preoperative patient selection – the tumour anatomy needs to be favourable such that resection is possible without damaging any major structures [35]. Techniques to control bleeding laparoscopically are discussed below.

*Oncologic resection –* A LLR should be aborted if a clear margin cannot be achieved. In contrast to the common adult hepatic tumours of colorectal metastases and HCC arising in the context of chronic liver disease, hepatoblastoma and *de novo* HCC in children are large tumours, often occupying a hemi-liver, making an R0 resection challenging and reducing the number of paediatric liver tumours that are amenable to LLR [24, 25, 36, 37].

### Surgical approach

*Patient position -* Ideal patient positioning depends on the size of the child. In older children, positioning in lithotomy favourable [3, 23, 38]. The surgeon stands between the patient’s legs and the assistant stands on the patient’s left [23, 38]. Left lateral decubitus is also an option if the tumour is in the right posterior section [38]. The patient may be kept level or placed in reverse Trendelenburg to allow viscera to fall away [38].

*Trocar sites-* One 10–12 mm trocar should be placed at the umbilicus [14, 23]. Generally, in smaller patients we will place 3 additional 5 mm trocars in line or just below the level of the umbilicus, while in older patients the working ports are placed above the umbilicus [25]. Ideal placement can be assessed after visualization of the field [14, 39]. An additional port can be added for retraction of the liver [25]. Specimen extraction is typically through the larger umbilical port or through an inguinal or suprapubic incision [25, 40].

For hand-assisted or laparoscopic-assisted procedures (hybrid), an additional incision is made, which can be used to palpate and explore the liver and lyse adhesions. It should be made below the xiphoid and kept as small as possible while allowing for specimen extraction or an inguinal incision can be utilized [14, 40].

In patients with portal hypertension, the umbilical port should be placed inferior to the umbilicus or superior to the umbilicus and lateral to the linea alba to avoid a recanalized umbilical vein [38]. Alternatively, the abdomen can be entered via the Hasson technique and the recanalized umbilical vein ligated.

### Surgical technique

#### Types

Laparoscopic options include the conventional 3–5 ports, laparoscopic hand-assisted and hybrid. The hand-assisted and hybrid options allow additional access for mobilization, retraction, rapid hemostasis and extraction [14]. However, unless operating on a large child or adolescent, the hand-assisted technique is unlikely to significantly advance the technical approach to LLR in children due to the limited surgical field [17]. In adults, a complete hepatectomy via a single incision has been performed, but this has not yet been reported in children [41].

Robotic-assisted hepatic surgery using the Da Vinci robot in adults is increasingly utilized [42]. While robotic surgery offers better visualization and separation of the tumour, its high cost, need for specialized instruments and limited training opportunities for paediatric surgeons have restricted its use in children [17, 26]. In paediatrics, the robot has been used for hepatic surgery more commonly with choledochal cyst excision and hepaticojejunostomies [43, 44]. There are a few case reports of robotic hepatectomies in children [26, 27, 45].

#### Steps

After trocar placement, intraoperative ultrasound can be used to demarcate resection margins as well as vascular structures [14]. The lesser sac should be accessed and the porta hepatis encircled; the remnant of the divided umbilical vein can be used to elevate the liver during exposure of the porta hepatis [22, 38].

There are different techniques for how to proceed with dissection. Once the liver is mobilized, the right or left artery, portal vein and hepatic duct can be exposed and divided [38]. Debate exists as to the best way to approach the hepatic vein, with different techniques described below. The line of resection should be decided based on either the line of demarcation or ultrasound [38]. The first 2–3 cm of Glisson’s capsule contain no major structures, so can be incised, then the parenchyma divided using either gross stapling technique or visualized dissection technique [38]. Options for transection include stapling, the CUSA, the harmonic scalpel and LigaSure^TM^ [14, 23, 25]. Glissonean pedicles should be ligated and divided with a knot-pusher or Hem-o-lock clip [39].

Alternatively, the Glissonean pedicle approach (Takasaki maneuver) can be utilized. This method involves ligation and division of the vessels at the hepatic hilum prior to segmentectomy. The Glissonean pedicle is dissected free and the extrahepatic segmental branch of the corresponding liver segment is divided. The tertiary branches supplying the segment can then be transected through a hilar or parenchymal approach [46].

Malignant lesions should be removed through a port in a bag with the lesion extended if needed, while benign lesions can be morcellated [14]. Drains are typically placed at the conclusion of the procedure [22].

### Tips and pitfalls

*General approach -* Case volume is central to governing the integration of LLR into a practice. Beginning with anatomically favourable cases allows for mastery of the technique. Living donor resections in adults can provide an opportunity for paediatric surgeons to perform LLR before transitioning to smaller patients with more friable livers [23]. While a recommendation is to complete 45–75 cases before performing a major hepatectomy, this number depends on prior experience. Additionally, the distinction between major and minor hepatectomy does not necessarily correlate with the difficulty of the procedure [36].

Instead of differentiating between major and minor, tumour location is key to choosing which procedure is suitable to perform via an MIS approach. Starting with a left lateral sectionectomy can lay the framework for success [22, 36]. Surgeons performing LLR should have experience with both hepatobiliary surgery and laparoscopic surgery [24].

While aspects of laparoscopic hepatectomy are similar to open hepatectomy, it is critical to adjust the perspective from which the liver is viewed. There are several recommendations for this. First, while in open liver resection, the view is anteroposterior, in LLR, the liver should be viewed from a caudal to cranial approach. This provides a better perspective of the liver hilum and roots of the hepatic veins and allows for upward dissection and transection of the liver parenchyma [37]. Second, the liver should be viewed as an open door in LLR versus an open book in open surgery [37]. Finally, major veins should be approached from the root rather than anteriorly [37].

### Step-specific

The traditional approach to transection of the parenchyma involves dividing the vasculature structures prior to dividing the lobe, with the resection then made at the zone of ischemia. Alternatively, the vessels can be divided as they are encountered, as is done with a wedge resection. The traditional approach has better vascular control but risks injury to hilar and retrohepatic structures during their dissection [38].

Using the gross stapling technique appears to decrease the risk of bleeding and lead to shorter operative time [22]. If utilizing the CUSA, the dissection can be done on a lower setting than in adults, given the healthy parenchyma of children (50% for aspiration, 3 mL/min for irrigation, 30% for amplitude, cavitation ++ in tissue select mode) [25]. In addition to avoiding damage to healthy parenchyma, a lower setting also causes less change in intrabdominal pressure, which preserves field visualization and less inflow of CO_2_ gas, thereby limiting tissue desiccation [25].

Parenchymal transection can also be completed with individual ligation of Glissonean structures [25]. However, this must be carefully performed given the shorter distance between pedicles as compared to adults [25]. Target vessels should have a distance of at least 7 mm between them to ensure the stump is long enough to avoid stricture [25]. If unable to create enough space between tumour and Glissonean structures or tumour and hepatic vein one technique involves applying a clip to the proximal side and manually clamping the distal side, then using one hand to transect the vessel and ligate the distal side with suture or coagulation [25]. This will prevent the use of force, which can lead to vascular injuries or bleeding from clips slipping off a short stump [25]. Lowering the intrabdominal pressure to 8 mmHg prior to dissecting the parenchyma decreases the risk of air embolism [22].

Indocyanine green (ICG) can be used to help define margins for oncologic resection [37]. It can also be used to evaluate the biliary tract, which is important for centrally located and hilar tumours and for delineation of liver segments [47]. Finally, the use of ICG during complex cases can be used for visualization of flow and to evaluate patency, kinking and stenosis of vessels [48]. A recommended dose is 0.3 mg/kg administered 48 hours before surgery [25].

### Achieving hemostasis and biliostasis

Direct pressure is the best method to control bleeding, which can be done manually if using the hand-assisted technique or with gauze pads [25, 49]. Small bleeders can be addressed with cautery, clips, stapler or hemostatic agents. Larger vessels should be stapled or suture ligated. Severe bleeding can be addressed through increased pneumoperitoneum and decreased airway pressure [25, 36]. If possible, the Pringle maneuver should be avoided as, in the more fragile paediatric liver, it can cause ischemic changes, portal vein thrombosis and damage to the hepatic artery [25]. If necessary, a temporary Pringle can be applied via an external tourniquet positioned through a 5 mm trocar [23]. Low central venous pressure anesthesia may also be used to help control bleeding [29, 38].

The argon cannot be used for biliostasis; this should be addressed with fibrin, cautery, saline-enhanced radiofrequency energy, clip or suture [25].

## MIS for radiofrequency ablation (RFA)

RFA is a management option for patients who cannot be rendered tumour free by resection alone. RFA is most often used in adults for HCC with metastases ≤3 cm as an alternative to resection or as a bridge to transplant [50, 51]. Although rarely used in children, several case series have been published that describe deploying this technique for paediatric patients with both benign and malignant liver lesions, including hepatoblastoma, HCC, adenoma, myofibroblastic tumour, metastases and infiltrative hepatic cysts [51–53]. The majority of patients experienced minimal complications and equivalent long-term survival compared to resection, suggesting that RFA should be considered as an option for the management of paediatric hepatic lesions in select patients [51–53]. RFA can be performed percutaneously, but on occasion may need laparoscopic assistance to allow access to lesions in the dome of the liver, where a percutaneous approach would traverse the diaphragm and potentially injure the lung.

### Indications

Hepatic RFA is indicated as either primary therapy for patients with small neoplasms, to shrink a mass in order to become amenable to resection or as a bridge to transplant [53, 54]. It has been successfully utilized in both the paediatric and adult population for primary and metastatic liver masses [51].

### Contraindications

Absolute contraindications to RFA include intravascular invasion, tumour location within 1 cm of the biliary duct, intrahepatic biliary tree dilation, exophytic location of the tumour or uncorrectable coagulopathy [55]. Although rare in children, end-stage cirrhosis (Child-Pugh C) is also an absolute contraindication [56]. Relative contraindications include extrahepatic metastases, bilioenteric fistula, lesions that are superficial or adjacent to the small intestine or gallbladder and platelets <50,000 /mm^3^ [55]. Additionally, RFA is unable to reliably destroy tumours ≥5 cm, so should not serve as primary therapy for larger tumours [57].

### Surgical approach

Tumour destruction with RFA is achieved by converting electrical current in the radiofrequency range into heat. This heat is passed through a closed-loop circuit consisting of the patient, needle electrodes, a generator and a grounding pad [56]. Success depends on maintaining the correct temperature and accurate targeting of the tumour [56].

RFA can be performed via a percutaneous or laparoscopic approach. A systemic review found that a laparoscopic approach has a higher rate of ablation success and fewer recurrences than a percutaneous approach, but also carries a higher rate of complications [58].

### Tips and pitfalls

Successful RFA is multifactorial, but a key component is accurate assessment of the hepatic tumour prior to the procedure [59]. This allows for optimizing the approach path and for appropriate caution when ablating subcapsular masses, which carry a higher risk of damage to surrounding organs [59]. Tumours that are small or recurrent isoechoic tumours also present a challenge, as they can be difficult to see on ultrasonography. These can be managed through follow-up imaging, use of contrast-enhanced ultrasonography or with transarterial chemoembolization in addition to RFA [59]. Additionally, tumours that are in close proximity to vasculature are at risk of losing heat to the vessels (‘heat-sink’) [57].

## Conclusion

MIS is an evolving technique in the toolbox of paediatric surgeons for the management of hepatic masses. Biopsy performed via MIS carries the standard advantages of a less invasive approach, as well as reducing adhesions and decreasing bleeding complications. Paediatric LLR in appropriate cases is technically feasible with the potential for shorter LOS, decreased blood loss, less pain and improved cosmesis. However, these factors should not take precedence over the need to perform a safe procedure that adheres to oncologic principles. Finally, RFA offers a chance at local disease control for patients who are otherwise not upfront surgical candidates due to tumour characteristics or patient comorbidities.

## Conflicts of interest

We have no conflicts of interest to disclose.

## Funding

We have no funding to declare.

## Figures and Tables

**Figure 1.**
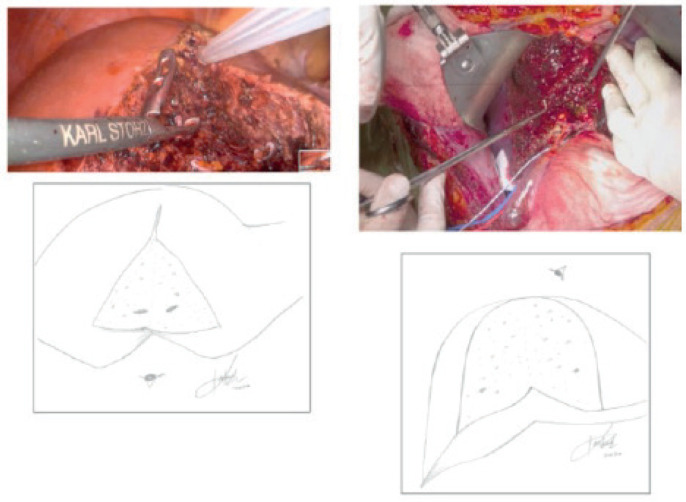
Images on the left display the laparoscopic ‘open-door’ view, while images on the right display the ‘open-book’ view utilized in open liver resection [34].

**Table 1.** Case reports describing minimally invasive liver resections for benign and malignant masses in paediatric patients.

Author	Published	Total number	Benign number	Malignant number	Age	Weight	Type of resection (*N*)	Outcomes	Follow-up time	Conversion to open	Complications
Yoon *et al* [22]	2006	1	1	0	5 years	NR	LLS	All alive in good clinical condition	10 months	0	0
Veenstra and Koffron [14]	2016	36	15	21	Med. 2.7 years	NR	Seg.* (10) Sec. (5) HH* (16)	No mortalities, recurrences, or reoperations	Med. 12 months	NR	5
Kwon *et al* [25]	2019	19	5	14	Med. 26 months	Med. 11 kg	PH (12) LLS (2) LL (1) RL (3) RPS (1)	Recurrence (1) Deceased, not from disease (1)	Med. 64 months	1	0
Chen *et al* [26]	2019	1	0	1	3 years	NR	S5 Sec.**	NR	NR	0	0
Murawski *et al* [3]	2021	6	6	0	4 months - 16 years	NR	BS (1) Wedge (5)	NR	NR	1	1
Sandlas *et al* [27]	2021	1	0	1	3 years	NR	RH	NR	NR	0	1
Larghi Laureiro *et al* [23]	2022	10	9	1	Med. 12 years	Med. 49.5 kg	Seg.(4) LLS (4) BS (1) PLS (1)	All alive in good clinical condition	24 months	2	3

(*) Proportion of cases performed via hand-assisted or hybrid laparoscopic approach. (**) Case performed robotically

Abbreviations:

BS: Bisegmentectomy: HH: Hemihepatectomy; Kg: Kilograms; LL: Left lobectomy; LLS: Left lateral sectionectomy; Med.: Median; NR: Not reported; PH: Partial hepatectomy; RH: Right hepatectomy; RL: Right lobectomy; RPS: Right posterior sectionectomy; Sec: Sectionectomy; Seg: Segmentectomy; PLS: Posterolateral sectionectomy
